# Effects of Postharvest Water Deficits on the Physiological Behavior of Early-Maturing Nectarine Trees

**DOI:** 10.3390/plants9091104

**Published:** 2020-08-27

**Authors:** María R. Conesa, Wenceslao Conejero, Juan Vera, M. Carmen Ruiz-Sánchez

**Affiliations:** Irrigation Department, CEBAS-CSIC. P.O. Box 164, Campus de Espinardo, 30100 Murcia, Spain; wenceslao@cebas.csic.es (W.C.); jvera@cebas.csic.es (J.V.); mcruiz@cebas.csic.es (M.C.R.-S.)

**Keywords:** midday stem water potential, *Prunus persica* L., transpiration efficiency, stomatal conductance, vapor pressure deficit, water deficit

## Abstract

The physiological performance of early-maturing nectarine trees in response to water deficits was studied during the postharvest period. Two deficit irrigation treatments were applied, moderate and severe, and these were compared with a control treatment (fully irrigated). Stem water potential and leaf gas exchange (net CO_2_ assimilation rate, A_CO2_; transpiration rate, E; and stomatal conductance, g_s_) were measured frequently. Drought avoidance mechanisms included a decrease in stomatal conductance, especially in the case of the severe deficit treatment, which also showed a strong dependence of A_CO2_ on g_s_. Intrinsic water-use efficiency (A_CO2_/g_s_) was more sensitive than instantaneous water-use efficiency (A_CO2_/E) as an indicator to detect water deficit situations in nectarine trees. However, in contrast to the results obtained for other deciduous fruit trees, a poor correlation was found between A_CO2_/E and A_CO2_/g_s_, despite the important relation between E and g_s_. A_CO2_/E was also weakly correlated with g_s_, although this relationship clearly improved when the vapor pressure deficit (VPD) was included, along with g_s_ as the independent variable. This fact reveals that apart from stomatal closure, E depends on the boundary layer conductance (g_b_), which is mediated by VPD through changes in wind speed. This suggests low values of the decoupling coefficient for this water-resilient species.

## 1. Introduction

Water scarcity in the semi-arid area of Spain is one of the most important environmental constrains affecting the physiology of crops. Its effects are expected to intensify as global temperatures increase [1,2,3]. Peaches and nectarines (*Prunus persica* L.) are one of the most common and economically important fruit tree species in the Mediterranean area, where nearly 70% of the rainfall is concentrated in autumn and with frequent drought periods during the growing season [4,5]. Spain is the fourth largest peach and nectarine producer with an average annual production of 1,42 Mt in the period 2015–2018 [6], acting as a leader in export to European markets. In early-maturing cultivars, fruit ripening coincides with periods of low crop evapotranspiration (ETc). Cultivation requires lower amounts of water than late-maturing cultivars, which is an important issue in semi-arid areas, where water is often a limiting factor for peach and nectarine production [4,5,6,7,8,9,10,11,12,13].

Deficit irrigation is frequently adopted to improve water-use efficiency and is considered an alternative to traditional irrigation scheduling approaches that fully meet plant water requirements [14]. Deficits are applied during the non-critical phenological periods when the sensitivity of the plant to water stress is minimal in terms of yield and quality. In early-maturing fruit trees, with a very short period from fruit set to harvest and a very long postharvest period, deficit irrigation should be applied only during the postharvest period [4,5,6,7,8,9,10,11,12,13]. Postharvest is an important period for nectarine fruits trees, since carbohydrates are accumulated and floral organ differentiation occurs [15,16], for which reason water deficits should be managed carefully at this time to avoid reductions in bloom and fruit load [4].

Plant physiological processes are directly affected by the amount of water that plants can extract from the soil [17]. Water stress limits plant growth and crop production more than any other environmental factor. In some cases, it may cause a deleterious output [18]. A reduction of stomatal conductance (g_s_) and leaf growth inhibition are among the first processes that plants develop in response to drought. They protect them from extensive water loss but reduce the source of assimilates (photosynthetic rate) and consequently affect the resulting biomass and final crop production [19]. Although minimizing water loss by stomatal closure under drought conditions clearly increases the water-use efficiency, it has also been seen to reduce CO_2_ uptake and leaf cooling via leaf transpiration [20,21,22,23]. Plants have developed many mechanisms to survive drought, including escape, tolerance, and avoidance to water deficits. Avoidance of stress includes changes in leaf area, anatomy, and orientation, among others, whereas tolerance to stress involves at least two mechanisms, osmotic adjustment and changes in the elastic properties of tissues [24,25,26]. Mellisho et al. [27] reported that drought resistance in early-maturing peach trees is based both on avoidance mechanisms such as stomatal control and tolerance mechanisms, including active osmotic adjustment and high apoplastic water content.

Reductions in leaf gas exchange parameters are often used as plant indicators of water deficit situations [22,28]. In order to better understand the mechanisms behind water stress when applying deficit irrigation strategies, the use of sensors in the soil–plant–atmosphere water continuum is compelled [1,11]. Previous studies have shown that continuous real-time measurements based on soil water content (θ_v_) with capacitance probes indicated accurately the impact of different irrigation events on the stored soil water. This provided useful information concerning the advance of the wetted front, the depth of the root system activity, and the fate of the applied water [4,5,6,7,8,9,10,11,12,13,14,15,16,17,18,19,20,21,22,23,24,25,26,27,28,29,30,31,32,33]. These θ_v_-based data allowed an optimal soil moisture range to be set for the crop. Then, it was possible to precisely adjust the irrigation dose and frequency and to automate irrigation [4,10]. In a companion paper by Conesa et al. [5], precise irrigation scheduling based on real-time information provided by soil moisture sensors was shown to be a promising tool. It allows water to be saved without compromising yield and fruit quality in early-maturing nectarine trees. However, the efficiency of the automated irrigation scheduling compared with conventional irrigation based on ETc has not been discussed in terms of physiological plant behavior until now.

Under field conditions, water stress is often accompanied by other environmental constraints, such as steep leaf-to-air water vapor gradients and high radiation and temperature, particularly in the Mediterranean regions [34]. The exposure of a single leaf or the whole plant to dry air is expected to increase transpiration because of the greater vapor pressure differences between the leaf and the air. However, such a situation may also decrease g_s_ and hence affect leaf transpiration. The concentration of water vapor inside the leaf changes with leaf temperature: as the temperature rises, the air may contain more water vapor, and subsequent evaporation from the wet surfaces of the leaf will raise the water vapor concentration to saturation, as has been found in leaves of both well-watered and water-stressed plants [35]. Stomatal closure responds with a non-lineal increase in transpiration rate to reach a plateau and, in some cases, to decrease if VPD levels are very high [36,37]. By preventing high transpiration rates, stomatal closure avoids the corresponding decline in plant water potential, protecting the plant from excessive dehydration and physiological damage. The response of stomatal conductance to increasing VPD is generally an exponential decrease, but the magnitude of the decrease—the stomatal sensitivity—varies considerably both within and between species. Stomatal sensitivity at low VPD is proportional to the magnitude of g_s_. Therefore, stomatal response to air humidity involves an apparent feed-forward response [38], because it enables the plant to restrict excessive water loss and may enhance the plant’s ability to use soil water supplies efficiently [20,21,22,23].

For these reasons, the main objective of this paper was to investigate to what extent changes in stomatal conductance (g_s_), in response to water deficits, are mediated by vapor pressure deficit (VPD) in early-maturing nectarine trees. Different levels of water deficit (moderate and severe) were applied during the postharvest period to early-maturing nectarine trees grown in a semi-arid area of Spain.

## 2. Results and Discussion

### 2.1. Water Applied and Meteorological Conditions

**Figure 1:**
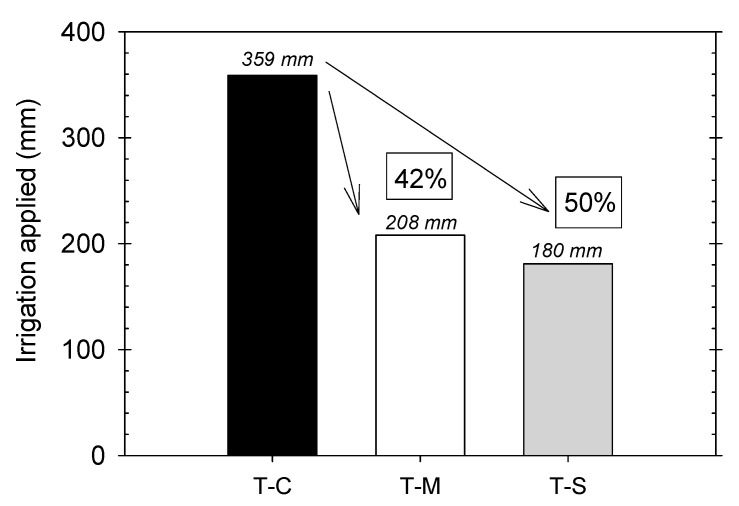
Irrigation applied (mm) during the postharvest period (May to October) in the different irrigation treatments. Percentage with respect to the Control treatment. (T–C). Moderate water deficit treatment (T–M); Severe water deficit treatment (T–S).

During the postharvest period, which comprised the experimental period, the average amount of water applied by irrigation to each treatment was 359, 208, and 180 mm in the T–C, T–M, and T–S water deficit treatments, respectively (Figure 1). In the T–M treatment, the soil water deficit imposed (based on a θ_v_ threshold value of α = 30%) represented a mean water reduction of about 42% compared with the T–C treatment, which was based on conventional ETc calculations. It should be noted that there was no penalty in yield and fruit quality as a result of this treatment [5]. Similarly, automated irrigation has been demonstrated as suitable efficient irrigation scheduling of early-maturing *Prunus* sp. trees [4,5,6,7,8,9,10,11,12,13,14,15,16,17,18,19,20,21,22,23]. In plum trees, the automated system based on soil moisture sensors proposed by Millán et al. [32] was able to establish a regulated deficit irrigation strategy based on 40% ETc. Moreover, the automated algorithm of Dominguez-Niño et al. [33] saved 23% of the irrigation volume compared with the traditional water balance.

Agro-meteorological conditions in the study area were typical for a Mediterranean climate with dry summers and mild–wet winters [17,39]. During the experimental period, rainfall amounted to 71 mm and ET_0_ 1017.6 mm (Table 1). Daily ET_0_ was highest in June, and September was the wettest month. VPD reached daily mean values ranging from −1.6 kPa in June to −0.6 kPa in October. Moreover, the mean wind speed and the global radiation decreased as the experiment progressed, with the highest values registered in May and June, respectively (Table 1). Considering the 10-year seasonal average rainfall and ET_0_ values (≈ 250 and 1320 mm, respectively [40]), the ET_0_ accumulated during the postharvest period represented 80% of the total water needs, whereas rainfall was low. 

### 2.2. Time Course of Plant–Water Relations

**Figure 2:**
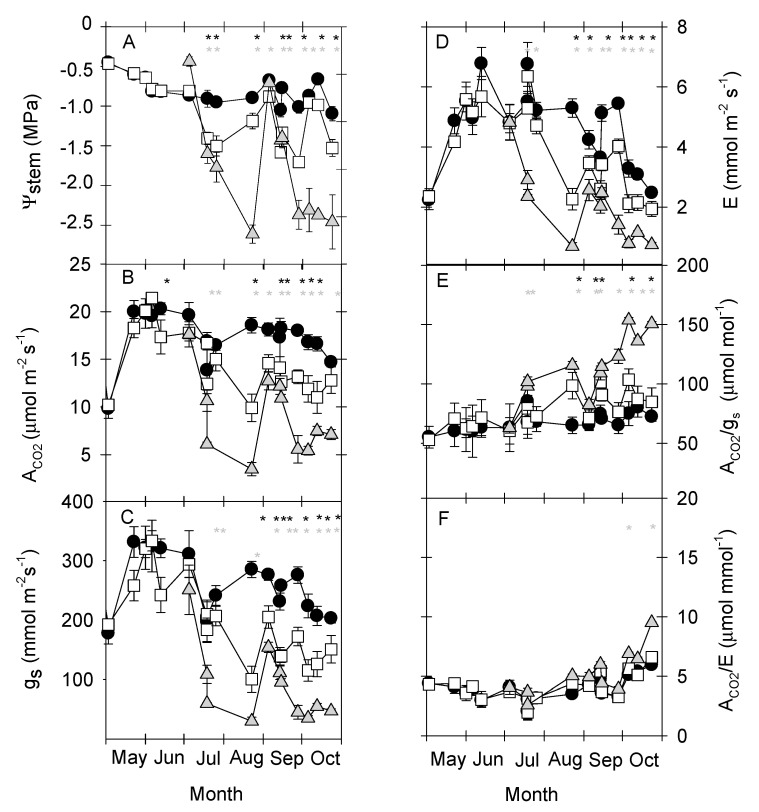
Seasonal trends of: (**A**) midday stem water potential (Ψ_stem_, MPa); (**B**) net CO_2_ assimilation rate (A_CO2_, µmol m^−2^ s^−1^); (**C**) stomatal conductance (g_s_, mmol m^−2^ s^−1^); (**D**) transpiration rate (E, mmol m^−2^ s^−1^); (**E**) intrinsic water-use efficiency (A_CO2_/g_s_, µmol mol^−1^); and (**F**) instantaneous water-use efficiency (A_CO2_/E, µmol mmol^−1^), in the different irrigation treatments: T–C (●), T–M (□), and T–S (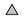). Each point is the mean ± standard error (n = 4). Asterisks indicate statistically significant differences between T–C and T–M (black) and T–S (gray) according to LSD_0.05_.

Regarding plant water status, the T–C treatment showed an average Ψ_stem_ value of about −0.80 MPa (Figure 2A), which is symptomatic of non-limiting soil water conditions in clay–loam soils [5,6,7,8,9,10,11,12,13,14,15,16,17,18,19,20,21,22,23,24,25,26,27,28,29,30,31,32,33,34,35,36,37,38,39,40,41,42]. In this sense, Abrisqueta et al. [30] affirmed that a Ψ_stem_ value above −0.9 MPa during the summer is an indicator of water stress initiation in early-maturing peach trees.

A non-flat pattern of the Ψ_stem_ in both deficit irrigation treatments was observed (Figure 2A), averaging −1.07 and −1.98 MPa in the T–M and T–S treatments, respectively. Differences with respect to the T–C treatment were constant and statistically significant from July onwards. The mean Ψ_stem_ difference from T–C was 0.27 MPa and 1.18 MPa for T–M and T–S treatments, respectively. Moreover, the greatest plant water deficits were registered during the late postharvest, with minimum Ψ_stem_ values of ≈ −1.71 and −2.68 MPa for T–M and T–S, respectively (Figure 2A).

Atmospheric-related effects on Ψ_stem_ (increased rainfall in August and September; Table 1, Figure 2A) emphasized the resilient nature of this *Prunus* species [43].

Values of the leaf gas exchange parameters (A_CO2_, g_s_ and E) were significantly reduced by water deficit as the experiment progressed, particularly in the T–S treatment (Figure 2B–D). In the T–C treatment, average values for A_CO2_, g_s_, and E were 17.5 µmol m^−2^ s^−1^, 261 mmol m^−2^ s^−1^, and 4.6 mmol m^−2^ s^−1^, respectively, which is characteristic of non-limiting soil water conditions, as indicated in Vera et al. [11] for early-maturing nectarine trees. The moderate water deficit imposed in the T–M treatment decreased the gas exchange season values by around 15%, 24%, and 15% for A_CO2_, g_s_, and E, respectively, compared with T–C values. As expected, withholding irrigation caused higher gas exchange reductions in the T–S treatment: 55% (for A_CO2_), 73% (for g_s_), and 63% (for E) compared with T–C values. Chaves et al. [19] reported that under mild to moderate water deficits, stomata closure is among the earliest of plant responses, restricting water loss and carbon assimilation. However, when water deficits intensified (as in the case of T–S treatment), the decreased gas exchange is motivated by the low rates of electron transport [44], as a result of a reduction in ATP synthesis [19,45].

The rainfall events in late summer (Table 1)were insufficient to enable the gas exchange values to reach the levels of the T–C treatment, in either T–M or T–S treatments, with their moderate and severe water deficit levels, respectively (Figure 2B–D). This relative delay in stomatal opening following re-watering is in contrast with the rapid recovery of the Ψ_stem_ (Figure 2A), as has also been observed in almond [22], apricot [46], citrus [24] trees, and table grapes [47], and it can be considered as a safety mechanism that allows plants to regain full turgor more efficiently [48].

The reductions in A_CO2_ and E associated with limited g_s_ are probably due to stomatal closure occurring when Ψ_stem_ declines below a threshold value [49,50]. In this sense, good lineal relationships were found between g_s_ and Ψ_stem_ [g_s_ = 333.63 + 116.32 Ψ_stem_, r^2^ = 0.55, *p* ≤ 0.001] and between A_CO2_ and Ψ_stem_ [A_CO2_ = 20.93 + 5.54 Ψ_stem_, r^2^ = 0.46; *p* ≤ 0.001] (data not shown). Rahmati et al. [36] observed a reduction (>50%) in leaf gas exchange when Ψ_stem_ decreased from −1.4 to −2.0 MPa in peach trees. However, an additional Ψ_stem_ decrease (≤−2.0 MPa) only led to a slight decrease in both g_s_ and A_CO2._ Shackel et al. [51] mentioned a Ψ_stem_ threshold value of ≈ −1.5 MPa when the decrease in A_CO2_ was compensated by the reduction in the vegetative apex growth. Those shoots are the major users of carbohydrates during the postharvest period, and they explain the summer pruning practices in deciduous fruit trees in an attempt to control excessive growth and alleviate the effect of water deficits [40,52].

**Figure 3:**
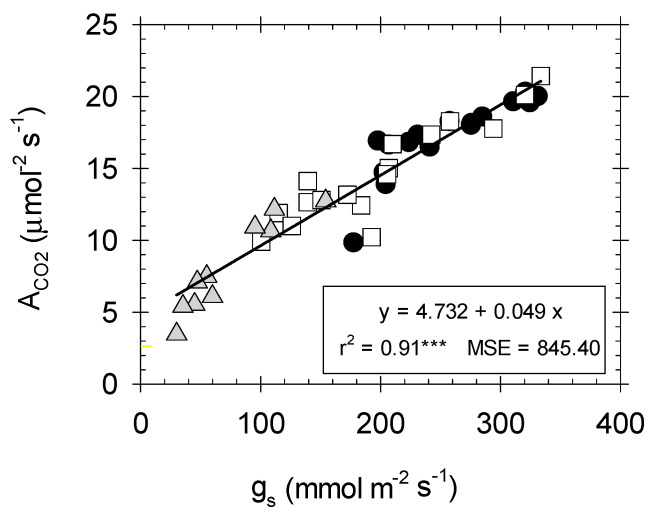
Relationship between stomatal conductance (g_s_, mmol m^−2^ s^−1^) and net CO_2_ assimilation rate (A_CO2_, µmol m^−2^ s^−1^) in the different irrigation treatments: T–C (●), T–M (□), and T–S (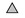). Each point is the mean of four leaves. Coefficient of determination for T–C (r^2^ = 0.71***), T–M (r^2^ = 0.83***), and T–S (r^2^ = 0.96***). ***: *p ≤* 0.001, MSE: mean squared error.

Values of intrinsic (A_CO2_/g_s_) and instantaneous water-use efficiency (A_CO2._/E) increased with water stress, reaching maximum values in the T–S treatment (Figure 3E,F). By closing the stomata, the water-use efficiency increases, reducing the amount of H_2_O lost per CO_2_ assimilated [20,21,22,23]. In this sense, Romero et al. [53] reported that A_CO2._/g_s_ and A_CO2_/E increased in deficit-irrigated vines up to Ψ_stem_ values of −1.3 to −1.4 MPa when g_s_ varied between 110 and 140 mmol m^−2^ s^−1^. Moreover, below these Ψ_stem_ threshold values, the leaf gas exchange efficiency did not increase, or it even dropped slightly. Interestingly, A_CO2_/g_s_ was more sensitive than A_CO2_/E as a plant water status indicator to detect deficit situations in early-maturing nectarine trees (Figure 3E,F).

### 2.3. Leaf Gas Exchange Relationships

Our results revealed a strong lineal dependence between A_CO2_ and g_s_ (r^2^ = 0.91, *p* ≤ 0.001) (Figure 3), which demonstrates the potential target of stomatal control of the photosynthetic process in the cultivar studied. Similar slopes of the individual relationships were evident from the analysis of covariance (data not shown). When all data were pooled, the coefficients of determination (r^2^) were seen to improve as the soil water deficit increased: T–S > T–M > T–C (Figure 3), which agrees with the observation that g_s,_ together with mesophyll conductance are key players in the photosynthesis process, determining the flux of CO_2_ that reaches the Rubisco carboxylation sites in the chloroplast stroma [54]. However, it is known that the role of g_s_ in the photosynthetic process is also related to the prevailing climatic factors [13]. Indeed, Galle et al. [55,56] found a certain degree of ‘uncoupling’ during drought acclimation and re-watering in herbaceous and woody plants.

**Figure 4:**
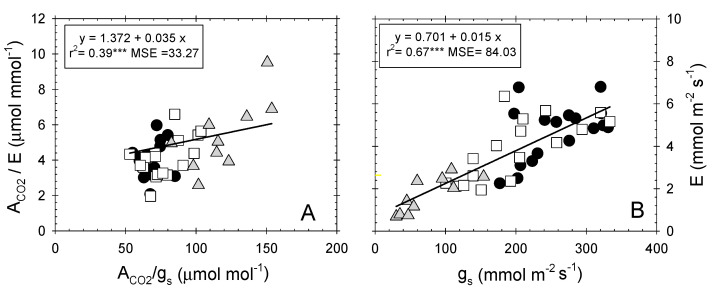
Relationship between (**A**) intrinsic water-use efficiency (A_CO2_/g_s_, µmol mol^−1^) and instantaneous water-use efficiency (A_CO2_/E, µmol mmol^−1^); and (**B**) stomatal conductance (g_s_, mmol m^−2^ s^−1^) and transpiration rate (E, mmol m^−2^ s^−1^) in the different irrigation treatments: T–C (●), T–M (□), and T–S (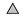). Each point is the mean of four leaves. Coefficient of determination in (**A**): T–C (r^2^ = 0.002 ns), T–M (r^2^ = 0.015 ns), and T–S (r^2^ = 0.52***); and in (**B**): T–C (r^2^ = 0.25***), T–M (r^2^ = 0.50***), and T–S (r^2^ = 0.70***). ns: not significant, ***: *p* ≤ 0.001, MSE: mean squared error.

A weak correlation was found between instantaneous (A_CO2_/E) and intrinsic (A_CO2_/g_s_) water-use efficiency (Figure 4A), despite the high dependence observed between E and g_s_ (Figure 4B) [57]. Nectarine trees behaved differently to table grape vines, in which high correlations between both water-use efficiencies (r^2^ = 0.88; *p* ≤ 0.001), as well as between E and g_s_ (r^2^ = 0.86; *p* ≤ 0.001), were described [47].

**Figure 5:**
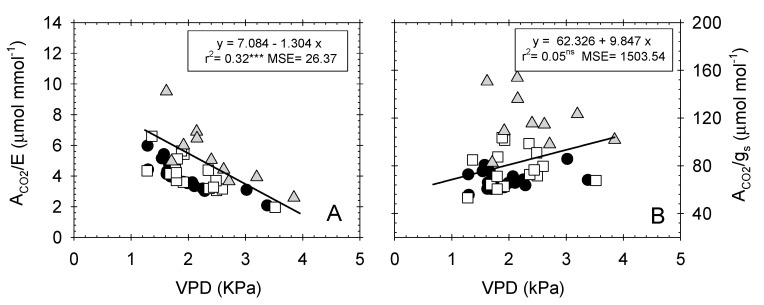
Relationship between vapor pressure deficit (VPD, kPa) and (**A**) instantaneous water-use efficiency (A_CO2_/E, µmol mmol^−1^); and (**B**) intrinsic water-use efficiency (A_CO2_/g_s_, µmol mol^−1^) in the different irrigation treatments: T–C (●), T–M (□), and T–S (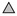). Each point is the mean of 4 leaves. Coefficient of determination in (**A**): T–C (r^2^ = 0.72***), T–S (r^2^ = 0.70***), T–S (r^2^ = 0.66*); and in (**B**): T–C (r^2^ = 0.15 ns), T–M (r^2^ = 0.10 ns), and T–S (r^2^ = 0.06 ns). ns: not significant, *: *p* ≤ 0.05, ***: *p* ≤ 0.001, MSE: mean squared error.

When both water-use efficiencies were correlated with the corresponding mean daily VPD values, a higher coefficient of determination was found for A_CO2_/E (r^2^ = 0.32 *p ≤* 0.001) (Figure 5A) than for A_CO2_/g_s_ (r^2^ = 0.05 ns) (Figure 5B). This could be explained by the fact that A_CO2_/E is more influenced by environmental conditions, since E depends on the degree of stomatal opening and the vapor pressure deficit (VPD) of the atmosphere surrounding the leaf [23,24,25,26,27,28,29,30,31,32,33,34,35,36,37,38,39,40,41,42,43,44,45,46,47,48,49,50,51,52,53,54,55,56,57,58], whereas A_CO2_/g_s_ excludes the effects of changing evaporative demand on water flux out of the leaf, and it depends only on the stomatal opening [59].

**Figure 6:**
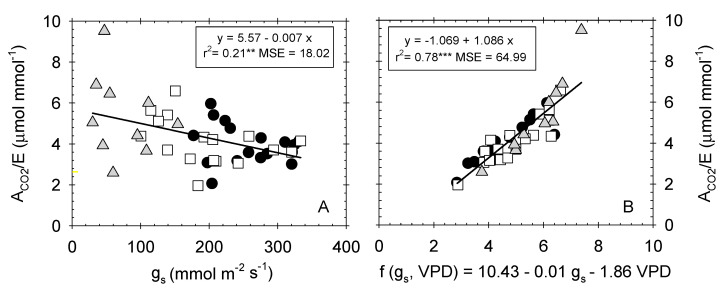
Relationship between (**A**) stomatal conductance (g_s_, mmol m^−2^ s^−1^) and instantaneous water-use efficiency (A_CO2_/E, µmol mmol^−1^); and (**B**) the two-variable function (g_s_, VPD) and A_CO2_/E in the different irrigation treatments: T–C (●), T–M (□), and T–S (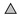). Each point is the mean of 4 leaves. Coefficient of determination in (**A**): T–C (r^2^ = 0.05 ns), T–S (r^2^ = 0.15 ns), T–S (r^2^ = 0. 06 ns); and in (**B**): T–C (r^2^ = 0.79***), T–M (r^2^ = 0.77***), and T–S (r^2^ = 0.85***). *: *p* ≤ 0.05 **: *p* ≤ 0.01, ***: *p* ≤ 0.001, MSE: mean squared error.

A poor degree of correlation was also noted between the A_CO2_/E and g_s_ (Figure 6A). Interestingly, when VPD data were included in the independent term along with g_s_, the coefficient of determination considerably improved (r^2^ = 0.78 *p* ≤ 0.001) (Figure 6B). This finding is explained because water loss from plant leaves is controlled not only by g_s_, but also by boundary layer conductance (g_b_), both operating in series [60]. The latter, g_b_, depends on the thickness of the layer of air at the surface of the leaf through which water vapor must diffuse after leaving the stomata [61]. Moreover, Martin et al. [60] reported that g_b_ is controlled by leaf size, morphology, and wind speed.

At this point, it should be remembered that the decoupling coefficient (Ω) (a dimensionless coefficient ranging from 0 to 1) represents the relative contribution of canopy stomatal (g_s_) and aerodynamic (g_b_) conductance in controlling rates of canopy transpiration [57,62]. Since g_b_ and g_s_ operate together, their relative magnitude determines which conductance is the dominant regulator of transpiration. Martin et al. [60] indicated that when g_s_ is much smaller than g_b_, stomata are the dominant controllers of water loss, and a decrease in g_s_ will result in a nearly proportional decrease in transpiration. Under this condition, the canopy and the atmosphere are fully aerodynamically coupled (Ω = 0), since E is controlled by the stomata conductance and VPD. In contrast, when g_b_ is much smaller than g_s_, changes in g_s_ will have little effect on E, and the input of radiation to the canopy will be the primary driver of leaf transpiration. In this state, the canopy and the atmosphere are fully aerodynamically decoupled (Ω = 1), since E is controlled by the energy balance.

Our results showed that changes in wind speed can modify VPD values through changes in air relative humidity, which would explain the low values of the decoupling coefficient (Ω) in early-maturing nectarine leaves. This emphasizes the advantage of introducing a meteorological variable along with a gas exchange parameter (as a two-variable function) for a better understating of the physiological behavior of plant leaves under water-deficit conditions.

## 3. Materials and Methods

### 3.1. Plant Material and Experimental Conditions

The experiment was performed from May to October 2017 in a 0.5 ha orchard of seven-year-old early-maturing nectarine trees (*Prunus persica* L. Batsch) cv. Flariba on GxN–15 rootstock, at the CEBAS–CSIC experimental station in Santomera, Murcia, Spain (38° 0,603,100 N, 1°0,201,400 W, 110 m altitude). Trees were spaced at 6.5 m × 3.5 m and trained to an open-center canopy. The soil in the 0–0.5 m layer was stony with a clay loam texture and low organic matter content. The average bulk density was 1.43 g cm^−3^. The volumetric soil water content (θ_v_) at the field capacity and permanent wilting point were 29% and 14%, respectively.

Crop management (including pest control) was that which was commonly used in commercial orchards in the area. Seasonal fertilizer applications were 100, 60, and 120 kg ha^−1^ of N, P_2_O_5_, and K_2_O, respectively, which were applied through a drip irrigation system [63]. The soil was kept free of weeds and was not tilled. Full bloom took place at the beginning of February; nectarine fruits were hand-thinned in March and harvested in early May. Nectarine trees were pruned annually during the dormancy period (mid-December).

Trees were drip irrigated with one line per tree row with four pressure-compensated emitters per tree each delivering 4 L h^−1^, located 0.5 and 1.3 m from the tree trunk. More details about the experimental site, soil and climate characteristics, fertilization, and cultural practices can be found elsewhere [5,6,7,8,9,10,11,12,13,14,15,16,17,18,19,20,21,22,23,24,25,26,27,28,29,30,31,32,33,34,35,36,37,38,39,40].

### 3.2. Irrigation Treatments

**Figure 7:**
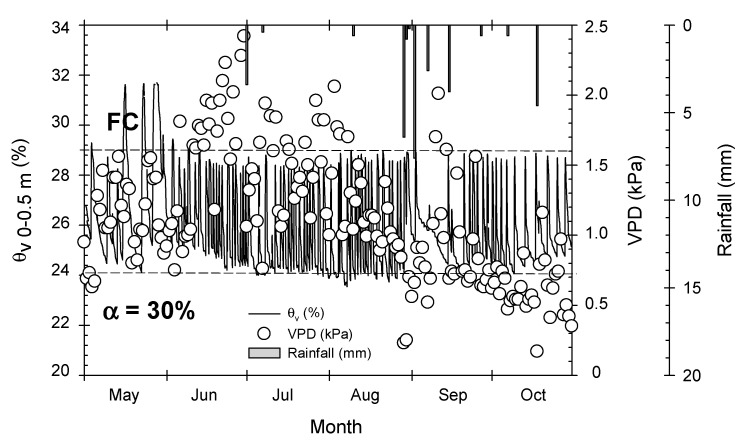
Soil water content (θ_v_, %) at 0–0.5 m soil depth during the postharvest period (May to October, 2017) in the moderate water deficit treatment (T–M). Values are the 15-min records corresponding to the average of four probes. Dashed horizontal lines delimit the field capacity (FC) and the soil water deficit (α) imposed. White points indicate daily vapor pressure values (VPD, kPa). Vertical bars indicate daily rainfall.

Three different irrigation treatments were applied:

- **Control treatment (T–C)**, fully irrigated throughout the growing season, and based on 100% of the crop evapotranspiration (ETc) to ensure non-limiting soil water conditions. ETc was estimated following the FAO approach [64] multiplying the crop reference evapotranspiration (ET_0_), using the Penman-Monteith equation [64], by the crop coefficients (Kc) obtained by Abrisqueta et al. [65] in the same location for *Prunus persica* sp. Irrigation was scheduled weekly, and the water was applied on a daily basis during the night as needed.

- **Moderate water deficit treatment (T–M),** based on reducing soil water content (automatically managed by means of soil water sensors) following the procedure indicated in Vera et al. [11]. Briefly, the volumetric soil water content (θ_v_) was measured at depths of 0.1, 0.3, 0.5, and 0.7 m using capacitance probes (EnviroScan^®^, Sentek Pty. Ltd., Adelaide, South Australia). One PVC access tube was installed 0.1 m from the emitter located 0.5 m from the trunk of four representative trees. The θ_v_ values in the 0–0.5 m soil profile, coinciding with the effective root depth [31], were computed and used to activate electro-hydraulic valves by means of a telemetry system. Threshold θ_v_ values were set to α = 30% to trigger irrigation and to the field capacity (FC) value to end irrigation (Figure 7) during the postharvest period (from May to October), the non-critical period of early-maturing *Prunus* sp. trees [4], while a lower α (10%) was applied during the critical period corresponding to the fruit growth period (March to May) [11].

- **Severe water deficit treatment (T–S),** which involved withholding irrigation during the late postharvest period (from August to October, 2017) and irrigating (100% of ETc), similarly to the T–C treatment during the rest of the growing season.

The experimental layout consisted of a completely randomized design with four replications per irrigation treatment, each consisting of six trees (the central four were used for measurements and the others served as guard trees), with a total of 24 trees per irrigation treatment. No active roots were seen more than 1.5 m from the drip line, as revealed in a root distribution study [66].

### 3.3. Measurements

Environmental data, including air temperature (T), relative humidity (RH), wind speed (u_2_), solar radiation, and rainfall were recorded following the World Meteorological Organization’s recommendations by an automated weather station located 0.25 m from the orchard in the same CEBAS-CSIC experimental field station (http://www.cebas.csic.es/general_spain/est_meteo.html), which read the values every 5 min and recorded the averages every 15 min. Crop reference evapotranspiration (ET_0_, FAO-56, Penman-Monteith) was calculated hourly [64]. Daily maximum, minimum, and mean air temperatures (T_max_, T_mean_, T_min_), and daily maximum, minimum, and mean relative humidity (RH_max_, RH_mean_, RH_min_) were calculated, and the daily mean vapor pressure deficit (VPD, kPa) was determined using the following equations:

(1) $$e^{o}\left(T\right)=0.6108*\exp\left[\frac{17.27*\;T}{T+237.3}\right]$$

(2) $$e_{s}=[e^{o}\left(T_{max}\right)+e^{o}\left(T_{min}\right)$$

(3) $$e_{a}=\;\frac{\left(T_{max}\right)\;*\;\frac{RH_{min}}{100}\;+\;\left(T_{min}\right)\;*\;\frac{RH_{max}}{100}}{2}$$

(4) $$VPD=e_{s}-e_{a}$$

where *e_s_* is the saturation vapor pressure, *e_a_* is the actual vapor pressure, *T* is the temperature (°C), and *RH* is the relative humidity (%) [64].

The volume of water applied in each irrigation treatment was measured by in-line water meters with digital output pulses (ARAD).

Tree water status was estimated by measuring midday stem water potential (Ψ_stem_) using a pressure chamber (Soil Moisture Equipment Corp. Model 3000). Measurements were taken at midday (≈12:00 h solar time) in one healthy mature leaf from each replicate tree of each irrigation treatment (n = 4). Leaves were selected from the north face of the tree, near the trunk, and placed in plastic bags covered with aluminum foil for at least 2 h prior to excision, following the recommendations of Hsiao [67] and McCutchan and Shackel [68]. Measurements were carried out every 7–10 days from May to October.

Leaf gas exchange measurements were made on the same days as Ψ_stem_, at around 10:00 h solar time, in one sun-exposed leaf per replicate and four replicates per irrigation treatment (n = 4). The net CO_2_ assimilation rate (A_CO2_, μmol m^−2^ s^−1^), stomatal conductance (g_s_, mmol m^−2^ s^−1^), and transpiration rate (E, mmol m^−2^ s^−1^) were measured at an ambient photosynthetic photon flux density (PPFD ≈ 1200 μmol m^−2^ s^−1^) and near-constant ambient CO_2_ concentration (Ca ≈ 400 μmol mol^−1^) with a field-portable closed photosynthesis system (LI-COR, LI-6400, Lincoln, NE, USA) equipped with a transparent 6 cm^2^ leaf chamber. From these parameters, the following parameters were obtained: intrinsic water-use efficiency, as the ratio between A_CO2_ and g_s_ (μmol mol^−1^) and instantaneous water-use efficiency, as the ratio between A_CO2_ and E (µmol mmol^−1^), which is also known as transpiration efficiency [50].

### 3.4. Statistical Analysis

The data were analyzed by one-way ANOVA using SPSS v 9.1 (IBM, Armonk, NY, USA) to discriminate between irrigation treatments. *Post hoc* pair-wise comparison between all means was performed by a Least Significant Difference (LSD) test at *p* ≤ 0.05 (LSD_0.05_). The degree of agreement of the regressions among variables was evaluated through the coefficient of determination (r^2^) and the mean squared error (MSE).

## 4. Conclusions

The results indicate that early-maturing nectarine trees are a resilient species that respond well to water stress, essentially by developing drought avoidance mechanisms. The water deficits applied during the postharvest period reduced plant water status and leaf gas exchange values, the most affected parameter being stomatal conductance. The T–M treatment, which was controlled by soil water content sensors, induced a moderate plant water deficit (Ψ_stem_ reduction of 0.27 MPa, with respect to the fully irrigated treatment, T–C), leading to a reduction in water application of up to 42% compared with T–C). Meanwhile, severely restricting irrigation (T–S) induced a greater plant water deficit (Ψ_stem_ reduction of 1.2 MPa with respect to T–C). Leaf transpiration, apart from stomatal closure, depends on the g_b_, which is mediated by VPD through changes in wind speed, modifying air relative humidity. We propose the inclusion of a meteorological variable, such as VPD, alongside a gas exchange parameter (as a two-variable function) for a better understanding of the physiological behavior of plant leaves under water deficit conditions, as this would better explain the changes that occur in transpiration efficiency (A_CO2_/E). The findings also point to low decoupling coefficient values (Ω) for early-maturing nectarine leaves, even though more research is needed in this respect.

## Figures and Tables

**Table 1:** Monthly values of total crop reference evapotranspiration (ET_0_), maximum, mean, and minimum air temperatures (T_max_, T_mean_, and T_min_), maximum, mean, and minimum relative humidity (RH_max_, RH_mean_, and RH_min_), mean vapor pressure deficit (VPD), total rainfall, and mean wind speed at 2 m (u_2_) and solar radiation.

	ET_0_ (mm)	Temperature (°C)			RH (%)			VPD (kPa)	Rainfall (mm)	u_2_ (km d^−1^)	Solar Radiation (W m^−2^)
Month		*T_max_*	*T_mean_*	*T_min_*	*RH_max_*	*RH_mean_*	*RH_min_*				
May	140.3	27	20.7	14.6	82.7	57.5	32.3	1.1	0	5.42	243.2
June	304.8	31.5	25.3	19.2	82.5	56.1	29.6	1.6	0	4.38	275.4
July	158.7	32.3	26.6	21	88.8	63.1	37.5	1.5	0.4	4.88	248.2
August	123	32.9	27.1	22	91.2	64.3	42.1	1.1	8	3.2	218.2
September	219.5	30	24.4	20.2	92	70.1	41.1	0.9	22.6	4.8	176.4
October	71.3	24	18.8	14.4	93.7	68.2	46.3	0.6	5.2	3.61	137.4
**Total/Mean**	1017.6	29.6	20.5	18.5	88.5	63.2	38.2	1.1	36.2	4.38	216.4
